# Anæsthetics in Cataract Operations

**Published:** 1884-08-20

**Authors:** 


**Anæsthetics in Cataract Operations.**—The improved methods of operating are mainly due to anæsthetics, and would not have been brought to their present perfection without them. It would certainly not be practicable, in a large proportion of conscious patients, to plan and execute an incision, within very accurately defined limits, at the summit of the cornea, for the simple reason that the instinctive tendency of the superior rectus to roll the eye upward and to remove it out of the way of injury would frequently be too strong to be resisted; while, if this tendency were overcome, against the efforts of the muscle, by forceps traction, there would be frequent laceration of the conjunctiva and there would be frequent loss of vitreous at quite an early period of the operation, from rupture of the hyaloid by the struggle between the forceps and the muscle, a struggle which may even now be witnessed, and which is not seldom attended by hurtful consequences, in cases in which the anæsthesia is incomplete. Still less would it have been possible, without the passive eye afforded by an anæsthetic, to carry out the series of almost experimental operations, of slight variations in the position and magnitude of the section, of which the procedure that I have endeavored to describe is the final outcome; so that, without anæsthetics, I do not believe that either the broad lines of the operation could have been successfully defined, or that those lines could have been strictly adhered to in practice. Against the great benefits of freedom from pain, from apprehension, from hurry and from restlessness of the eye, there is, according to my experience, only a single counterbalancing disadvantage, namely, the harm which may possibly arise from obstinate vomiting. I have only seen one instance, already referred to, in which the effects of such vomiting were destructive. I have seen many in which vomiting of moderate severity has been productive of no ill result whatever.—*Braithwaite's Retrospect.*

---

Truth and love are two of the most powerful things in the world, and when they both go together they cannot easily be withstood.

